# *P*,*P*-Diphenyl-*N*-(1,1,2,2-tetra­phenyl-1λ^5^-diphosphanyl­idene)phosphinous amide

**DOI:** 10.1107/S1600536808020412

**Published:** 2008-07-09

**Authors:** Diane A. Dickie, Richard A. Kemp

**Affiliations:** a Department of Chemistry and Chemical Biology, MSC03 2060, 1 University of New Mexico, Albuquerque, NM 87131, USA

## Abstract

The title compound, C_36_H_30_NP_3_, a structural isomer of tris­(diphenyl­phosphino)amine, was unexpectedly isolated as the sole phospho­rus-containing product from the reaction of Mg[N(PPh_2_)_2_]_2_(THF)_2_ (THF is tetra­hydro­furan) with CO_2_. Its identity was confirmed by ^31^P NMR spectroscopy and single-crystal X-ray diffraction. The geometry at the two P(III) atoms is trigonal pyramidal, while the P(V) atom adopts a distorted tetrahedral geometry.

## Related literature

For the original synthesis and spectroscopic characterization of the title compound, see: Nöth & Meinel (1967 ▶); Meinel & Nöth (1970 ▶). For the crystallographic characterization of the structural isomer N[P(C_6_H_5_)_2_]_3_, see: Ellermann *et al.* (1987 ▶). For related literature, see: Bruno *et al.* (2004 ▶).
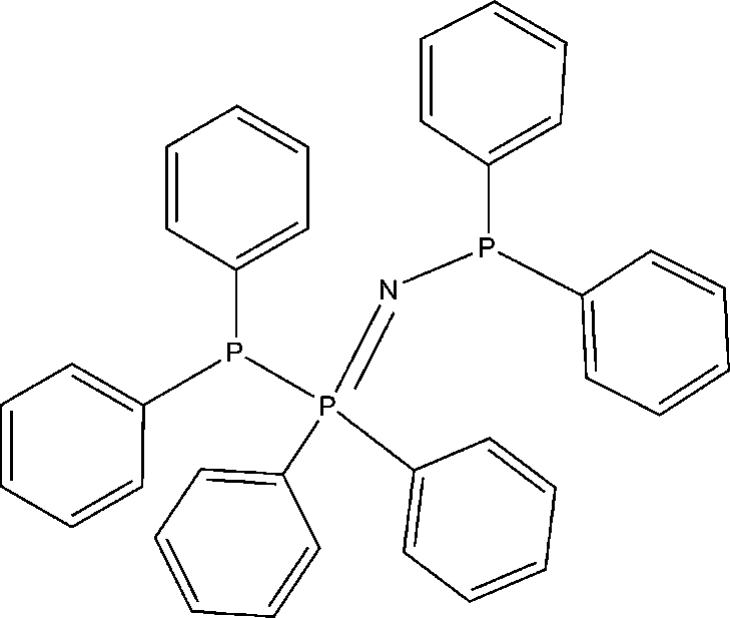


## Experimental

### 

#### Crystal data

C_36_H_30_NP_3_
*M* *_r_* = 569.52
Monoclinic, 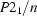

*a* = 9.3026 (13) Å
*b* = 10.8167 (15) Å
*c* = 29.750 (4) Å
β = 98.589 (6)°
*V* = 2960.0 (7) Å^3^
*Z* = 4
Mo *K*α radiation
μ = 0.23 mm^−1^
*T* = 228 (2) K
0.57 × 0.51 × 0.18 mm

#### Data collection

Bruker SMART CCD area-detector diffractometer
Absorption correction: multi-scan (*SADABS*; Bruker, 2001 ▶) *T*_min_ = 0.88, *T*_max_ = 0.96
76797 measured reflections
11496 independent reflections
9167 reflections with *I* > 2σ(*I*)
*R*_int_ = 0.029

#### Refinement

*R*[*F* ^2^ > 2σ(*F* ^2^)] = 0.042
*wR*(*F* ^2^) = 0.118
*S* = 1.05
11496 reflections
481 parameters
All H-atom parameters refined
Δρ_max_ = 0.41 e Å^−3^
Δρ_min_ = −0.29 e Å^−3^

### 

Data collection: *SMART* (Bruker, 2003 ▶); cell refinement: *SAINT* (Bruker, 2004 ▶); data reduction: *SAINT*; program(s) used to solve structure: *SHELXS97* (Sheldrick, 2008 ▶); program(s) used to refine structure: *SHELXL97* (Sheldrick, 2008 ▶); molecular graphics: *DIAMOND* (Brandenburg, 1999 ▶); software used to prepare material for publication: *publCIF* (Westrip, 2008 ▶).

## Supplementary Material

Crystal structure: contains datablocks I, global. DOI: 10.1107/S1600536808020412/at2581sup1.cif

Structure factors: contains datablocks I. DOI: 10.1107/S1600536808020412/at2581Isup2.hkl

Additional supplementary materials:  crystallographic information; 3D view; checkCIF report

## Figures and Tables

**Fig. 1.**
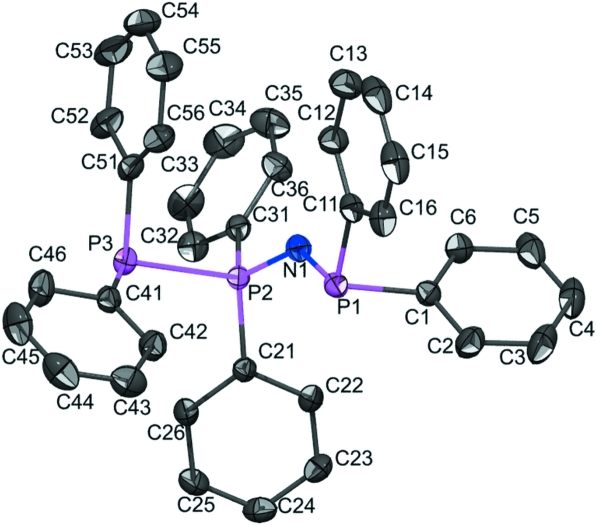
View of the title compound showing full numbering scheme. Ellipsoids are shown at 50% probability and hydrogen atoms have been removed for clarity.

## supplementary crystallographic information

### Comment

The molecular stucture of the title compound, (I), is shown in Fig. 1. It was originally prepared by Nöth and Meinel [Nöth & Meinel (1967); and Meinel & Nöth (1970)] but its crystal structure was not determined at that time. We report herein the isolation of (I) as an unexpected product of the reaction of carbon dioxide with Mg[N(PPh_2_)_2_]_2_(THF)_2_. This compound was characterized by ^31^P NMR spectroscopy and single-crystal X-ray diffraction. The P═N double bond measures 1.5690 (10) Å, very close to the average value of similar bonds in a *Mogul* (Bruno *et al.*, 2004) search of the Cambridge structural database (mean P=N 1.573 Å). The P—N single bond of 1.6755 (11) Å is significantly shorter than those in the structural isomer N(PPh_2_)_3_ (Ellermann, *et al.* 1987) (mean P—N = 1.740 Å), which is not surprising when the different hybridization of nitrogen (*sp*^2^*versus sp*^3^) is considered. It is also, however, shorter than the average P—N(*sp*^2^) bond length of 1.706 Å. The geometry at each of the two P(III) atoms is trigonal pyramidal, due to the stereochemically active lone pair on each of these atoms. The P(V) atom adopts distorted tetrahedral geometry.

### Experimental

Under an inert argon atmosphere, Mg[N(PPh_2_)_2_]_2_(THF)_2_ (0.67 g, 0.72 mmol) was dissolved in 40 ml anhydrous THF. The solution was exposed to 2 eq. of carbon dioxide at 10 psig. After 16 h, the solution was purged with argon. Colourless crystals of the title compound crystallized from the solution over the course of two weeks. ^31^P{^1^H} NMR (101.255 MHz, THF) δ 41.5 (d, ^2^J_PP_ = 97 Hz, Ph_2_*P*-N), 17.8 (d of d, ^2^J_PP_ = 97 Hz, ^1^J_PP_ = 249 Hz, N=*P*Ph_2_), -9.4 (d, ^1^J_PP_ = 249 Hz, P-*P*Ph_2_) p.p.m..

### Refinement

H atoms were located from a difference Fourier map and refined isotropically.

### Crystal data

**Table:**

C_36_H_30_NP_3_	*F*_000_ = 1192
*M**_r_* = 569.52	*D*_x_ = 1.278 Mg m^−^^3^
Monoclinic, *P*2_1_/*n*	Mo *K*α radiation λ = 0.71073 Å
Hall symbol: -P 2yn	Cell parameters from 9384 reflections
*a* = 9.3026 (13) Å	θ = 2.3–33.2º
*b* = 10.8167 (15) Å	µ = 0.23 mm^−^^1^
*c* = 29.750 (4) Å	*T* = 228 (2) K
β = 98.589 (6)º	Square, colourless
*V* = 2960.0 (7) Å^3^	0.58 × 0.51 × 0.18 mm
*Z* = 4	

### Data collection

**Table:**

Bruker SMART CCD area-detector diffractometer	11496 independent reflections
Radiation source: fine-focus sealed tube	9167 reflections with *I* > 2σ(*I*)
Monochromator: graphite	*R*_int_ = 0.029
*T* = 228(2) K	θ_max_ = 33.5º
φ and ω scans	θ_min_ = 2.4º
Absorption correction: multi-scan(SADABS; Bruker, 2001)	*h* = −14→14
*T*_min_ = 0.88, *T*_max_ = 0.96	*k* = −16→16
76797 measured reflections	*l* = −45→45

### Refinement

**Table:**

Refinement on *F*^2^	Secondary atom site location: difference Fourier map
Least-squares matrix: full	Hydrogen site location: inferred from neighbouring sites
*R*[*F*^2^ > 2σ(*F*^2^)] = 0.042	All H-atom parameters refined
*wR*(*F*^2^) = 0.118	*w* = 1/[σ^2^(*F*_o_^2^) + (0.0543*P*)^2^ + 1.05*P*] where *P* = (*F*_o_^2^ + 2*F*_c_^2^)/3
*S* = 1.06	(Δ/σ)_max_ = 0.001
11496 reflections	Δρ_max_ = 0.41 e Å^−^^3^
481 parameters	Δρ_min_ = −0.29 e Å^−^^3^
Primary atom site location: structure-invariant direct methods	Extinction correction: none

### Special details

**Table:**

Geometry. All e.s.d.'s (except the e.s.d. in the dihedral angle between two l.s. planes) are estimated using the full covariance matrix. The cell e.s.d.'s are taken into account individually in the estimation of e.s.d.'s in distances, angles and torsion angles; correlations between e.s.d.'s in cell parameters are only used when they are defined by crystal symmetry. An approximate (isotropic) treatment of cell e.s.d.'s is used for estimating e.s.d.'s involving l.s. planes.
Refinement. Refinement of *F*^2^ against ALL reflections. The weighted *R*-factor *wR* and goodness of fit *S* are based on *F*^2^, conventional *R*-factors *R* are based on *F*, with *F* set to zero for negative *F*^2^. The threshold expression of *F*^2^ > σ(*F*^2^) is used only for calculating *R*-factors(gt) *etc*. and is not relevant to the choice of reflections for refinement. *R*-factors based on *F*^2^ are statistically about twice as large as those based on *F*, and *R*- factors based on ALL data will be even larger.

### Fractional atomic coordinates and isotropic or equivalent isotropic displacement parameters (Å^2^)

**Table:**

	*x*	*y*	*z*	*U*_iso_*/*U*_eq_	
N1	0.89827 (12)	0.55872 (10)	0.13502 (4)	0.0285 (2)	
P1	0.83490 (3)	0.52397 (3)	0.080793 (10)	0.02539 (7)	
P2	0.98888 (3)	0.67406 (3)	0.154676 (10)	0.02169 (6)	
P3	0.88674 (3)	0.85071 (3)	0.171889 (11)	0.02562 (7)	
C1	0.92572 (12)	0.37613 (11)	0.07254 (4)	0.0265 (2)	
C2	0.94010 (18)	0.34039 (15)	0.02865 (5)	0.0398 (3)	
H2	0.915 (2)	0.396 (2)	0.0046 (7)	0.052 (5)*	
C3	0.9911 (2)	0.22253 (18)	0.02040 (7)	0.0533 (4)	
H3	0.998 (3)	0.200 (2)	−0.0104 (8)	0.067 (7)*	
C4	1.03047 (18)	0.14200 (15)	0.05566 (7)	0.0494 (4)	
H4	1.062 (2)	0.061 (2)	0.0493 (7)	0.064 (6)*	
C5	1.02013 (17)	0.17767 (14)	0.09955 (7)	0.0433 (3)	
H5	1.045 (2)	0.120 (2)	0.1238 (7)	0.059 (6)*	
C6	0.96912 (15)	0.29430 (13)	0.10817 (5)	0.0344 (3)	
H6	0.958 (2)	0.3171 (19)	0.1389 (7)	0.053 (6)*	
C11	0.65810 (12)	0.45178 (11)	0.08595 (4)	0.0270 (2)	
C12	0.60725 (15)	0.43744 (13)	0.12725 (5)	0.0339 (3)	
H12	0.661 (2)	0.4670 (17)	0.1541 (6)	0.039 (5)*	
C13	0.47230 (17)	0.38278 (15)	0.12874 (6)	0.0444 (3)	
H13	0.437 (2)	0.380 (2)	0.1578 (7)	0.055 (6)*	
C14	0.38678 (16)	0.34312 (14)	0.08938 (7)	0.0468 (4)	
H14	0.297 (2)	0.306 (2)	0.0912 (7)	0.063 (6)*	
C15	0.43625 (16)	0.35656 (14)	0.04816 (7)	0.0439 (4)	
H15	0.378 (2)	0.332 (2)	0.0199 (7)	0.062 (6)*	
C16	0.57122 (15)	0.41024 (13)	0.04646 (5)	0.0349 (3)	
H16	0.6072 (19)	0.4179 (17)	0.0181 (6)	0.037 (4)*	
C21	1.13511 (12)	0.72160 (10)	0.12433 (4)	0.02325 (19)	
C22	1.20846 (14)	0.63055 (12)	0.10377 (5)	0.0318 (2)	
H22	1.180 (2)	0.5479 (18)	0.1056 (6)	0.045 (5)*	
C23	1.32338 (15)	0.66224 (14)	0.08106 (5)	0.0371 (3)	
H23	1.372 (2)	0.597 (2)	0.0673 (7)	0.057 (6)*	
C24	1.36496 (15)	0.78400 (14)	0.07826 (5)	0.0358 (3)	
H24	1.448 (2)	0.8079 (19)	0.0628 (6)	0.049 (5)*	
C25	1.29177 (17)	0.87515 (14)	0.09808 (6)	0.0420 (3)	
H25	1.318 (2)	0.957 (2)	0.0961 (7)	0.061 (6)*	
C26	1.17755 (16)	0.84426 (12)	0.12116 (6)	0.0371 (3)	
H26	1.132 (2)	0.909 (2)	0.1355 (7)	0.055 (6)*	
C31	1.06971 (12)	0.63888 (11)	0.21231 (4)	0.0256 (2)	
C32	1.16599 (14)	0.72246 (13)	0.23711 (5)	0.0326 (2)	
H32	1.197 (2)	0.7950 (18)	0.2228 (6)	0.042 (5)*	
C33	1.21154 (17)	0.70324 (17)	0.28318 (5)	0.0419 (3)	
H33	1.277 (2)	0.7602 (18)	0.2992 (6)	0.045 (5)*	
C34	1.16275 (19)	0.60144 (19)	0.30440 (5)	0.0488 (4)	
H34	1.191 (2)	0.587 (2)	0.3358 (7)	0.056 (6)*	
C35	1.0706 (2)	0.51676 (18)	0.27995 (6)	0.0491 (4)	
H35	1.041 (2)	0.449 (2)	0.2946 (7)	0.061 (6)*	
C36	1.02326 (16)	0.53572 (13)	0.23390 (5)	0.0362 (3)	
H36	0.953 (2)	0.4794 (18)	0.2163 (6)	0.047 (5)*	
C41	0.77800 (13)	0.90632 (11)	0.11987 (4)	0.0268 (2)	
C42	0.78720 (15)	0.86532 (13)	0.07605 (5)	0.0334 (3)	
H42	0.842 (2)	0.7942 (18)	0.0716 (6)	0.041 (5)*	
C43	0.71842 (18)	0.93088 (16)	0.03862 (5)	0.0420 (3)	
H43	0.727 (2)	0.9039 (19)	0.0095 (7)	0.052 (5)*	
C44	0.64070 (18)	1.03665 (16)	0.04451 (6)	0.0470 (4)	
H44	0.597 (2)	1.082 (2)	0.0194 (7)	0.058 (6)*	
C45	0.6267 (2)	1.07566 (16)	0.08773 (7)	0.0506 (4)	
H45	0.578 (3)	1.150 (2)	0.0926 (8)	0.070 (7)*	
C46	0.69448 (17)	1.01113 (14)	0.12517 (6)	0.0406 (3)	
H46	0.684 (3)	1.038 (2)	0.1553 (8)	0.069 (7)*	
C51	0.75546 (14)	0.78245 (12)	0.20420 (4)	0.0296 (2)	
C52	0.78649 (17)	0.79087 (16)	0.25158 (5)	0.0407 (3)	
H52	0.878 (3)	0.839 (2)	0.2671 (8)	0.067 (7)*	
C53	0.6955 (2)	0.73483 (19)	0.27841 (6)	0.0523 (4)	
H53	0.719 (3)	0.744 (2)	0.3110 (8)	0.073 (7)*	
C54	0.5732 (2)	0.67193 (19)	0.25838 (7)	0.0581 (5)	
H54	0.512 (3)	0.633 (2)	0.2772 (8)	0.071 (7)*	
C55	0.5397 (2)	0.66580 (18)	0.21142 (7)	0.0523 (4)	
H55	0.460 (2)	0.623 (2)	0.1988 (7)	0.058 (6)*	
C56	0.63044 (15)	0.72019 (14)	0.18414 (5)	0.0375 (3)	
H56	0.608 (2)	0.7168 (18)	0.1505 (6)	0.044 (5)*	

### Atomic displacement parameters (Å^2^)

**Table:**

	*U*^11^	*U*^22^	*U*^33^	*U*^12^	*U*^13^	*U*^23^
N1	0.0314 (5)	0.0258 (4)	0.0285 (5)	−0.0062 (4)	0.0052 (4)	−0.0046 (4)
P1	0.02921 (14)	0.02131 (13)	0.02575 (14)	−0.00134 (10)	0.00446 (11)	0.00031 (10)
P2	0.02190 (12)	0.01984 (12)	0.02357 (13)	0.00031 (9)	0.00413 (9)	−0.00128 (9)
P3	0.02697 (13)	0.02346 (13)	0.02645 (14)	0.00363 (10)	0.00405 (11)	−0.00289 (10)
C1	0.0255 (5)	0.0250 (5)	0.0293 (5)	−0.0026 (4)	0.0054 (4)	−0.0024 (4)
C2	0.0473 (8)	0.0408 (7)	0.0325 (7)	0.0040 (6)	0.0102 (6)	−0.0065 (6)
C3	0.0592 (10)	0.0503 (10)	0.0526 (10)	0.0056 (8)	0.0156 (8)	−0.0225 (8)
C4	0.0397 (7)	0.0323 (7)	0.0766 (12)	0.0038 (6)	0.0104 (8)	−0.0161 (7)
C5	0.0372 (7)	0.0297 (6)	0.0628 (10)	0.0048 (5)	0.0063 (7)	0.0041 (6)
C6	0.0359 (6)	0.0307 (6)	0.0374 (7)	0.0035 (5)	0.0083 (5)	0.0025 (5)
C11	0.0249 (5)	0.0220 (5)	0.0331 (6)	0.0018 (4)	0.0009 (4)	−0.0004 (4)
C12	0.0308 (6)	0.0335 (6)	0.0378 (7)	−0.0013 (5)	0.0066 (5)	0.0010 (5)
C13	0.0349 (7)	0.0404 (8)	0.0605 (10)	−0.0025 (6)	0.0159 (7)	0.0065 (7)
C14	0.0275 (6)	0.0296 (6)	0.0824 (13)	−0.0023 (5)	0.0047 (7)	0.0017 (7)
C15	0.0327 (6)	0.0313 (7)	0.0621 (10)	0.0017 (5)	−0.0115 (6)	−0.0075 (6)
C16	0.0333 (6)	0.0302 (6)	0.0383 (7)	0.0035 (5)	−0.0038 (5)	−0.0041 (5)
C21	0.0229 (4)	0.0222 (5)	0.0249 (5)	0.0011 (4)	0.0042 (4)	−0.0002 (4)
C22	0.0330 (6)	0.0257 (5)	0.0389 (7)	0.0029 (4)	0.0133 (5)	−0.0014 (5)
C23	0.0362 (6)	0.0369 (7)	0.0418 (7)	0.0059 (5)	0.0179 (6)	−0.0011 (5)
C24	0.0294 (6)	0.0439 (7)	0.0362 (7)	0.0003 (5)	0.0112 (5)	0.0068 (5)
C25	0.0428 (7)	0.0304 (6)	0.0570 (9)	−0.0060 (6)	0.0216 (7)	0.0032 (6)
C26	0.0395 (6)	0.0231 (5)	0.0533 (8)	−0.0019 (5)	0.0214 (6)	−0.0032 (5)
C31	0.0246 (5)	0.0256 (5)	0.0265 (5)	0.0029 (4)	0.0035 (4)	0.0013 (4)
C32	0.0288 (5)	0.0358 (6)	0.0319 (6)	−0.0015 (5)	0.0006 (5)	−0.0008 (5)
C33	0.0358 (7)	0.0542 (9)	0.0329 (7)	0.0022 (6)	−0.0042 (5)	−0.0036 (6)
C34	0.0501 (9)	0.0644 (11)	0.0297 (7)	0.0090 (8)	−0.0009 (6)	0.0087 (7)
C35	0.0574 (9)	0.0501 (9)	0.0395 (8)	0.0004 (8)	0.0065 (7)	0.0178 (7)
C36	0.0414 (7)	0.0321 (6)	0.0347 (7)	−0.0024 (5)	0.0043 (5)	0.0063 (5)
C41	0.0270 (5)	0.0227 (5)	0.0306 (5)	0.0023 (4)	0.0044 (4)	0.0014 (4)
C42	0.0363 (6)	0.0321 (6)	0.0314 (6)	0.0050 (5)	0.0039 (5)	0.0009 (5)
C43	0.0453 (8)	0.0467 (8)	0.0322 (7)	−0.0005 (6)	−0.0002 (6)	0.0070 (6)
C44	0.0443 (8)	0.0412 (8)	0.0506 (9)	0.0016 (6)	−0.0088 (7)	0.0160 (7)
C45	0.0508 (9)	0.0344 (7)	0.0634 (11)	0.0167 (7)	−0.0021 (8)	0.0064 (7)
C46	0.0440 (7)	0.0319 (7)	0.0451 (8)	0.0143 (6)	0.0045 (6)	−0.0007 (6)
C51	0.0309 (5)	0.0318 (6)	0.0275 (5)	0.0088 (4)	0.0089 (4)	0.0001 (4)
C52	0.0431 (7)	0.0515 (9)	0.0290 (6)	0.0117 (6)	0.0105 (6)	0.0003 (6)
C53	0.0608 (10)	0.0637 (11)	0.0370 (8)	0.0184 (9)	0.0226 (7)	0.0118 (7)
C54	0.0637 (11)	0.0529 (10)	0.0678 (12)	0.0132 (8)	0.0425 (10)	0.0185 (9)
C55	0.0424 (8)	0.0508 (9)	0.0690 (12)	−0.0034 (7)	0.0252 (8)	0.0009 (8)
C56	0.0329 (6)	0.0409 (7)	0.0408 (7)	0.0017 (5)	0.0118 (5)	−0.0006 (6)

### Geometric parameters (Å, °)

**Table:**

N1—P2	1.5690 (10)	C24—H24	0.99 (2)
N1—P1	1.6755 (11)	C25—C26	1.3892 (19)
P1—C1	1.8421 (12)	C25—H25	0.92 (2)
P1—C11	1.8476 (12)	C26—H26	0.95 (2)
P2—C31	1.8071 (12)	C31—C36	1.3882 (18)
P2—C21	1.8156 (11)	C31—C32	1.4023 (18)
P2—P3	2.2273 (5)	C32—C33	1.388 (2)
P3—C51	1.8196 (13)	C32—H32	0.958 (19)
P3—C41	1.8200 (13)	C33—C34	1.379 (3)
C1—C2	1.3873 (18)	C33—H33	0.94 (2)
C1—C6	1.3935 (19)	C34—C35	1.385 (3)
C2—C3	1.395 (2)	C34—H34	0.95 (2)
C2—H2	0.94 (2)	C35—C36	1.390 (2)
C3—C4	1.371 (3)	C35—H35	0.92 (2)
C3—H3	0.96 (2)	C36—H36	0.99 (2)
C4—C5	1.379 (3)	C41—C42	1.3917 (18)
C4—H4	0.95 (2)	C41—C46	1.3965 (18)
C5—C6	1.385 (2)	C42—C43	1.393 (2)
C5—H5	0.96 (2)	C42—H42	0.943 (19)
C6—H6	0.97 (2)	C43—C44	1.378 (2)
C11—C12	1.3895 (18)	C43—H43	0.93 (2)
C11—C16	1.3972 (18)	C44—C45	1.378 (3)
C12—C13	1.394 (2)	C44—H44	0.93 (2)
C12—H12	0.933 (18)	C45—C46	1.384 (2)
C13—C14	1.382 (3)	C45—H45	0.95 (2)
C13—H13	0.97 (2)	C46—H46	0.96 (2)
C14—C15	1.380 (3)	C51—C56	1.398 (2)
C14—H14	0.93 (2)	C51—C52	1.3991 (19)
C15—C16	1.391 (2)	C52—C53	1.386 (2)
C15—H15	0.97 (2)	C52—H52	1.04 (2)
C16—H16	0.957 (17)	C53—C54	1.382 (3)
C21—C22	1.3909 (16)	C53—H53	0.97 (2)
C21—C26	1.3915 (17)	C54—C55	1.387 (3)
C22—C23	1.3901 (18)	C54—H54	0.96 (2)
C22—H22	0.94 (2)	C55—C56	1.386 (2)
C23—C24	1.379 (2)	C55—H55	0.91 (2)
C23—H23	0.97 (2)	C56—H56	0.990 (19)
C24—C25	1.379 (2)		
			
P2—N1—P1	129.01 (7)	C24—C25—C26	120.13 (13)
N1—P1—C1	102.74 (6)	C24—C25—H25	120.2 (14)
N1—P1—C11	101.80 (6)	C26—C25—H25	119.7 (14)
C1—P1—C11	94.35 (5)	C25—C26—C21	120.58 (12)
N1—P2—C31	108.08 (6)	C25—C26—H26	118.3 (13)
N1—P2—C21	116.14 (5)	C21—C26—H26	121.1 (13)
C31—P2—C21	107.15 (5)	C36—C31—C32	119.56 (12)
N1—P2—P3	122.96 (4)	C36—C31—P2	119.62 (10)
C31—P2—P3	95.39 (4)	C32—C31—P2	120.40 (9)
C21—P2—P3	104.38 (4)	C33—C32—C31	119.97 (13)
C51—P3—C41	104.57 (6)	C33—C32—H32	119.6 (11)
C51—P3—P2	96.68 (4)	C31—C32—H32	120.4 (11)
C41—P3—P2	106.86 (4)	C34—C33—C32	119.90 (15)
C2—C1—C6	118.77 (13)	C34—C33—H33	121.4 (12)
C2—C1—P1	118.55 (10)	C32—C33—H33	118.7 (12)
C6—C1—P1	122.45 (10)	C33—C34—C35	120.53 (15)
C1—C2—C3	120.23 (16)	C33—C34—H34	121.2 (13)
C1—C2—H2	119.5 (13)	C35—C34—H34	118.3 (13)
C3—C2—H2	120.2 (13)	C34—C35—C36	120.01 (15)
C4—C3—C2	120.34 (16)	C34—C35—H35	119.2 (14)
C4—C3—H3	121.6 (14)	C36—C35—H35	120.8 (14)
C2—C3—H3	118.0 (15)	C31—C36—C35	120.00 (14)
C3—C4—C5	119.92 (15)	C31—C36—H36	118.7 (11)
C3—C4—H4	119.2 (13)	C35—C36—H36	121.3 (11)
C5—C4—H4	120.9 (13)	C42—C41—C46	118.52 (12)
C4—C5—C6	120.29 (16)	C42—C41—P3	125.94 (9)
C4—C5—H5	119.1 (13)	C46—C41—P3	114.86 (10)
C6—C5—H5	120.5 (13)	C41—C42—C43	120.11 (13)
C5—C6—C1	120.40 (14)	C41—C42—H42	120.0 (11)
C5—C6—H6	119.7 (12)	C43—C42—H42	119.8 (11)
C1—C6—H6	119.8 (12)	C44—C43—C42	120.54 (15)
C12—C11—C16	118.50 (12)	C44—C43—H43	119.9 (13)
C12—C11—P1	123.05 (10)	C42—C43—H43	119.6 (13)
C16—C11—P1	118.45 (10)	C45—C44—C43	119.81 (14)
C11—C12—C13	120.16 (14)	C45—C44—H44	119.6 (13)
C11—C12—H12	120.7 (11)	C43—C44—H44	120.6 (13)
C13—C12—H12	119.1 (11)	C44—C45—C46	120.09 (15)
C14—C13—C12	120.79 (16)	C44—C45—H45	121.3 (14)
C14—C13—H13	121.1 (12)	C46—C45—H45	118.4 (14)
C12—C13—H13	117.9 (13)	C45—C46—C41	120.86 (15)
C15—C14—C13	119.60 (14)	C45—C46—H46	120.0 (14)
C15—C14—H14	121.1 (13)	C41—C46—H46	119.1 (14)
C13—C14—H14	119.3 (13)	C56—C51—C52	119.65 (13)
C14—C15—C16	119.93 (15)	C56—C51—P3	123.44 (10)
C14—C15—H15	121.8 (13)	C52—C51—P3	116.88 (11)
C16—C15—H15	118.3 (13)	C53—C52—C51	119.98 (17)
C15—C16—C11	121.02 (15)	C53—C52—H52	119.4 (12)
C15—C16—H16	120.3 (11)	C51—C52—H52	120.6 (12)
C11—C16—H16	118.6 (11)	C54—C53—C52	120.06 (16)
C22—C21—C26	118.84 (11)	C54—C53—H53	122.1 (15)
C22—C21—P2	118.12 (9)	C52—C53—H53	117.8 (15)
C26—C21—P2	123.03 (9)	C53—C54—C55	120.31 (16)
C23—C22—C21	120.17 (12)	C53—C54—H54	119.3 (14)
C23—C22—H22	120.4 (12)	C55—C54—H54	120.4 (14)
C21—C22—H22	119.4 (12)	C56—C55—C54	120.30 (18)
C24—C23—C22	120.53 (12)	C56—C55—H55	120.5 (14)
C24—C23—H23	121.4 (13)	C54—C55—H55	119.2 (14)
C22—C23—H23	118.1 (13)	C55—C56—C51	119.66 (16)
C23—C24—C25	119.74 (12)	C55—C56—H56	121.4 (11)
C23—C24—H24	121.5 (12)	C51—C56—H56	118.9 (11)
C25—C24—H24	118.7 (12)		
